# Short-term efficacy and safety of enfortumab vedotin plus pembrolizumab as a first-line treatment for advanced urothelial carcinoma regardless of cisplatin eligibility

**DOI:** 10.1093/jjco/hyag117

**Published:** 2026-07-20

**Authors:** Takuji Hayashi, Atsunari Kawashima, Yoshiyuki Yamamoto, Yu Ishizuya, Hiromu Horitani, Shunsuke Inoguchi, Yuki Horibe, Nesrine Sassi, Yujiro Hayashi, Takeshi Ujike, Kosuke Nakano, Osamu Miyake, Hitoshi Takayama, Koji Yazawa, Kenji Tsutsui, Yutaka Kurahashi, Wataru Nakata, Toshihisa Asakura, Tetsuya Murakawa, Koichi Okada, Masashi Nakayama, Taigo Kato, Koji Hatano, Norio Nonomura

**Affiliations:** Department of Urology, The University of Osaka Graduate School of Medicine, 2-2, Yamadaoka, Suita, Osaka 565-0871, Japan; Department of Urology, The University of Osaka Graduate School of Medicine, 2-2, Yamadaoka, Suita, Osaka 565-0871, Japan; Department of Urology, The University of Osaka Graduate School of Medicine, 2-2, Yamadaoka, Suita, Osaka 565-0871, Japan; Department of Urology, The University of Osaka Graduate School of Medicine, 2-2, Yamadaoka, Suita, Osaka 565-0871, Japan; Department of Urology, The University of Osaka Graduate School of Medicine, 2-2, Yamadaoka, Suita, Osaka 565-0871, Japan; Department of Urology, The University of Osaka Graduate School of Medicine, 2-2, Yamadaoka, Suita, Osaka 565-0871, Japan; Department of Urology, The University of Osaka Graduate School of Medicine, 2-2, Yamadaoka, Suita, Osaka 565-0871, Japan; Department of Urology, The University of Osaka Graduate School of Medicine, 2-2, Yamadaoka, Suita, Osaka 565-0871, Japan; Department of Urology, Osaka International Cancer Institute, Osaka, Osaka, 3-1-69 Otemae, Chuo-ku, Osaka 540-0008, Japan; Department of Urology, Osaka Rosai Hospital, Sakai, Osaka, 1179-3 Nagasonecho, Kita-ku, Sakai 591-8025, Japan; Department of Urology, Osaka General Medical Center, Osaka, Osaka, 3-1-56 Bandaihigaishi, Sumiyoshi-ku, Osaka 558-8558, Japan; Department of Urology, Toyonaka Municipal Hospital, Toyonaka, Osaka, 4-14-1 Shibaharacho, Toyonaka 560-8565, Japan; Department of Urology, Sakai City Medical Center, Sakai, Osaka, 1-1-1 Ebaraji-cho, Nishi-ku, Sakai 593-8304, Japan; Department of Urology, Osaka Keisatsu Hospital, Osaka, Osaka, 2-6-40 Karasugatsuji, Tennoji-ku, Osaka 543-0042, Japan; Department of Urology, Minoh City Hospital, Minoh, Osaka, 5-7-1 Kayano, Minoh 562-0014, Japan; Department of Urology, Hyogo Prefectural Nishinomiya Hospital, Nishinomiya, Hyogo, 13-9 Rokutanji-cho, Nishinomiya, 662-0918, Japan; Department of Urology, Ikeda City Hospital, Ikeda, Osaka, 3-1-14 Jonan, Ikeda 563-8510, Japan; Department of Urology, Nippon Life Hospital, Osaka, Osaka, 2-1-54 Enokojima, Nishi-ku, Osaka 550-0006, Japan; Department of Urology, Higashiosaka City Medical Center, Higashiosaka, Osaka, 3-4-5 Nishiiwata, Higashiosaka 578-8588, Japan; Department of Urology, National Hospital Organization Osaka National Hospital, Osaka, Osaka, 2-1-14 Hoenzaka, Chuo-ku, Osaka 540-0006, Japan; Department of Urology, Osaka International Cancer Institute, Osaka, Osaka, 3-1-69 Otemae, Chuo-ku, Osaka 540-0008, Japan; Department of Urology, The University of Osaka Graduate School of Medicine, 2-2, Yamadaoka, Suita, Osaka 565-0871, Japan; Department of Urology, The University of Osaka Graduate School of Medicine, 2-2, Yamadaoka, Suita, Osaka 565-0871, Japan; Department of Urology, The University of Osaka Graduate School of Medicine, 2-2, Yamadaoka, Suita, Osaka 565-0871, Japan

**Keywords:** urothelial carcinoma, first-line therapy, enfortumab vedotin plus pembrolizumab, real-world practice, cisplatin eligibility

## Abstract

**Objective:**

This study aimed to evaluate the short-term efficacy and safety of enfortumab vedotin plus pembrolizumab (EV + P) as a first-line systemic therapy for patients with advanced urothelial carcinoma (UC) in real-world practice, with a focus on cisplatin (CDDP) eligibility.

**Methods:**

Seventy-six patients who received EV + P treatment for advanced UC at 13 institutions between November 2024 and March 2025 were retrospectively included in this study. We collected the clinical characteristics and treatment data of the patients and assessed treatment response, progression-free survival (PFS), and adverse events (AEs). Patients were divided into two groups based on CDDP eligibility, and both efficacy and safety were compared between the groups.

**Results:**

The median observation period was 5.56 (IQR, 4.77–6.21) months. Complete response was achieved in 6 (7.9%) patients receiving EV + P, partial response in 40 (52.6%), stable disease in 20 (26.3%), and progressive disease in 10 (13.2%). Six-month and median PFS rates were 80.6% and not reached, respectively. AEs of any grade and grade ≥3 occurred in 59 (77.6%) and 25 (32.9%) patients, respectively. The most frequently observed AE was skin disorders, which occurred in 36 (47.4%) patients; no grade 5 AEs were observed. Treatment response, PFS, and the cumulative incidence of AEs did not differ between CDDP-ineligible and CDDP-eligible patients.

**Conclusions:**

The short-term efficacy of EV + P treatment seemed comparable to that in previous reports, and the safety profile was acceptable, regardless of CDDP eligibility.

## Teaser

Short-term efficacy of EV + P treatment for advanced UC demonstrated comparable to that in previous reports, and safety profile was acceptable, regardless of CDDP eligibility.

## Introduction

Urothelial carcinoma (UC) arises from the urothelial endothelium, encompassing the upper (renal pelvis and ureter) and lower (bladder and urethra) urinary tract. Patients with metastatic or unresectable UC have a very poor prognosis [1, 2]. The 5-year survival rate of patients with metastatic bladder cancer is <10% [1].

Cisplatin (CDDP)-based chemotherapies are recommended as first-line treatment for patients with metastatic or unresectable UC. Although gemcitabine plus CDDP (GC) chemotherapy has been a first-line regimen until recent years [3], patients with renal dysfunction, Eastern Cooperative Oncology Group (ECOG) performance status (PS) of ≥2, audiometric hearing loss, peripheral neuropathy, or heart failure are considered ineligible for CDDP [4]. Pembrolizumab, an immune checkpoint inhibitor (ICI), has been used as first-line treatment in CDDP-ineligible patients with advanced UC; however, despite longer durations of response, it has lower response rates in a limited proportion of these patients [5]. In contrast, enfortumab vedotin (EV), an antibody–drug conjugate (ADC), has demonstrated a high response rate in patients with advanced UC, even after chemotherapy and ICI treatment [6].

Recently, a large-scale randomized phase III trial revealed that EV plus pembrolizumab (EV + P) demonstrated better outcomes than conventional chemotherapy in CDDP-ineligible and CDDP-eligible patients with previously untreated UC [7]. We have been using this combination therapy in Japan since November 2024 as an insurance-covered treatment for patients with advanced UC. However, beyond clinical trials, few reports of EV + P treatment have been published [8, 9].

This study aimed to evaluate the short-term efficacy and safety of EV + P as a first-line systemic therapy for patients with advanced UC in real-world practice, with a focus on CDDP eligibility as a patient-related factor.

## Patients and methods

### Study design

We retrospectively included 76 patients who received EV + P treatment for advanced UC at The University of Osaka Hospital and 12 affiliated institutions between November 2024 and March 2025 without predefined exclusion criteria. Metastasis was diagnosed on the basis of imaging findings in most patients, whereas some cases were pathologically confirmed by biopsy. All patients received 1.25 mg/kg of EV intravenously on days 1 and 8 and 200 mg of pembrolizumab on day 1 of a 21-day cycle. Dose modifications or discontinuation of either agent was performed at the discretion of the treating physician. This study was approved by the Institutional Review Board of Osaka University Hospital (No. 23530).

Clinical characteristics and treatment data were collected from the medical records. Continuous variables are presented as median and interquartile range (IQR). CDDP eligibility was defined as an estimated glomerular filtration rate ≥ 50 mL/min/1.73 m^2^ and ECOG PS ≤1. Local treatment was defined as either surgical resection or radiotherapy directed at the primary tumor. Adverse events (AEs) were graded according to the Common Terminology Criteria for Adverse Events, version 6.0. Hypothyroidism and adrenal insufficiency were defined as immune-related endocrinopathies. Cases with missing data were excluded from the relevant analyses, and no imputation was performed. Median observation period was estimated using the reverse Kaplan–Meier method.

Treatment response was classified as the best overall response based on imaging and clinical evaluations using the Response Evaluation Criteria in Solid Tumors, version 1.1, conducted under routine clinical practice at each institution; these included complete response (CR), partial response (PR), stable disease (SD), or progressive disease (PD). The objective response rate (ORR) comprised CR and PR, and the disease control rate (DCR) included CR, PR, and SD. All data were presented as median (IQR) or number (%). The cumulative incidence of each AE, dose modifications of EV, progression-free survival (PFS), and overall survival (OS) were calculated using the Kaplan–Meier method.

Dose modification of EV was defined as “dose reduction or treatment discontinuation of EV due to AEs.” Cumulative incidence of each AE and dose modification of EV was calculated using the Kaplan–Meier method. Patients were censored at treatment discontinuation due to disease progression. Minimum dose of EV was defined as the lowest dose reached after dose reduction caused by AEs during treatment, but not by disease progression, to better characterize the safety profile.

Patients were divided into two groups (CDDP-eligible and CDDP-ineligible), and efficacy and safety were compared between the groups.

### Statistical analysis

The log-rank test was used for between-group comparisons of PFS, the cumulative incidence of each AE, and dose modifications. Statistical analyses were performed using JMP Pro 15.0.0 (SAS Institute Inc., Cary, NC, USA), and *P* < .05 was considered statistically significant.

## Results

### Clinical characteristics and treatment data

The clinical characteristics of the 76 patients are presented in Table 1. The median age was 74 years (IQR, 66–81 years), and 54 (71.1%) patients were male. A total of 39 (51.3%), 29 (38.2%), and 8 (10.5%) patients had an ECOG PS of 0, 1, and ≥2, respectively. Thirty-five (46.1%) patients were eligible for CDDP, whereas 41 (53.9%) were ineligible. Regarding the histological type, 57 (75.0%) patients had only UC, and 14 (18.4%) had a morphologic subtype or divergent differentiation. Primary lesions were in the renal pelvis or ureter in 27 (35.5%) patients, bladder or urethra in 44 (57.9%), and both in 5 (6.6%). Metastatic lesions comprised the lymph node alone in 25 (32.9%) patients, peritoneal dissemination or local recurrence in 16 (21.1%), lung in 17 (22.4%), bone in 12 (15.8%), and liver in 7 (9.2%).

**Table 1:** Patient characteristics and treatment data (*n* = 76).

		Total (*n* = 76)	Eligible (*n* = 35)	Ineligible (*n* = 41)	*P*-value
Age (years), median (IQR)		74 (66–81)	73 (62–78)	77 (69–83)	.033^a^
Sex *n* (%)	Male	54 (71.1)	21 (60.0)	33 (80.5)	.0496^a^
	Female	22 (28.9)	14 (40.0)	8 (19.5)	
ECOG PS *n* (%)	0	39 (51.3)	22 (62.9)	17 (41.5)	–
	1	29 (38.2)	13 (37.1)	16 (39.0)	
	≥ 2	8 (10.5)	0 (0.0)	8 (19.5)	
Histologic subtype *n* (%)	UC only	57 (75.0)	26 (74.3)	31 (75.6)	.356
	Morphologic subtype or divergent differentiation	14 (18.4)	8 (22.8)	6 (14.6)	
	Unknown	5 (6.6)	1 (2.9)	4 (9.8)	
Primary lesion *n* (%)	Renal pelvis, ureter	27 (35.5)	7 (20.0)	20 (48.8)	.007^a^
	Bladder, urethra	44 (57.9)	27 (77.1)	17 (41.4)	
	Both	5 (6.6)	1 (2.9)	4 (9.8)	
Metastatic lesion *n* (%)	LN only	25 (32.9)	12 (34.3)	13 (31.7)	.812
	Lung	17 (22.4)	5 (14.3)	12 (29.3)	.118
	Bone	12 (15.8)	4 (11.4)	8 (19.5)	.335
	Liver	7 (9.2)	2 (5.7)	5 (12.2)	.330
History of local treatment *n* (%)	Yes	38 (50.0)	14 (40.0)	24 (58.5)	.107
	No	38 (50.0)	21 (60.0)	17 (41.5)	
History of perioperative treatment *n* (%)	Yes	22 (28.9)	11 (31.4)	11 (26.8)	.659
	No	54 (71.1)	24 (68.6)	30 (73.2)	
History of chemotherapy *n* (%)	Yes	21 (27.6)	10 (28.6)	11 (26.8)	.866
	No	55 (72.4)	25 (71.4)	30 (73.2)	
History of ICI therapy *n* (%)	Yes	2 (2.6)	2 (5.7)	0 (0.0)	.121
	No	74 (97.4)	33 (94.3)	41 (100.0)	
Premedication with steroids *n* (%)	Yes	62 (81.6)	24 (68.6)	38 (92.7)	.007^a^
	No	14 (18.4)	11 (31.4)	3 (7.3)	
Initial dose reduction *n* (%)	Yes	4 (5.3)	1 (2.9)	3 (7.3)	.386
	No	72 (94.7)	34 (97.1)	38 (92.7)	
AE *n* (%)	Yes ≤G2/≥G3	59 (77.6) 34 (44.7)/25 (32.9)	26 (74.3) 15 (42.9)/11 (31.4)	33 (80.5) 19 (46.4)/14 (34.1)	.518 .790/0.802
	No	17 (22.4)	9 (25.7)	8 (19.5)	–
Current medication *n* (%)	Ongoing	48 (63.2)	22 (62.9)	26 (63.4)	.429
	Discontinuation AE/progression	28 (36.8) 18 (23.7)/10 (13.1)	13 (37.1) 10 (28.6)/3 (8.5)	15 (36.6) 8 (19.5)/7 (17.1)	–
Observation period (month), median (IQR)		5.56 (4.77–6.21)	6.67 (4.87–7.50)	4.50 (3.62–5.75)	.015^a^

IQR, interquartile range; CDDP, cisplatin; ECOG, Eastern Cooperative Oncology Group; eGFR, estimated glomerular filtration rate; LN, lymph node; PS, performance status; UC, urothelial carcinoma; ICI, immune checkpoint inhibitor; AE, adverse event; EV, enfortumab vedotin.

^a^CDDP eligibility: eligible was defined as eGFR ≥50.0 mL/min/1.73 m^2^ and PS ≤1.

Treatment data are also summarized in Table 1. A total of 38 (50.0%), 22 (28.9%), 21 (27.6%), and 2 (2.6%) patients had a history of local treatment, perioperative treatment, chemotherapy, and ICI therapy, respectively. Premedication with steroids and initial dose reductions were administered to 62 (81.6%) and 4 (5.3%) patients, respectively. AEs of any grade, grade ≤ 2, and grade ≥ 3 occurred in 59 (77.6%), 34 (44.7%), and 25 (32.9%) patients, respectively. EV + P was ongoing in 48 (63.2%) patients, whereas EV + P was discontinued owing to AEs in 18 (23.7%) patients and disease progression in 10 (13.1%). The median observation period was 5.56 (IQR, 4.77–6.21) months.

### Efficacy of EV + P treatment

The treatment responses to EV + P were CR in 6 (7.9%) patients, PR in 40 (52.6%), SD in 20 (26.3%), and PD in 10 (13.2%) (Fig. 1a). The ORR was 60.5%, and DCR was 86.8%.

**Figure 1:**
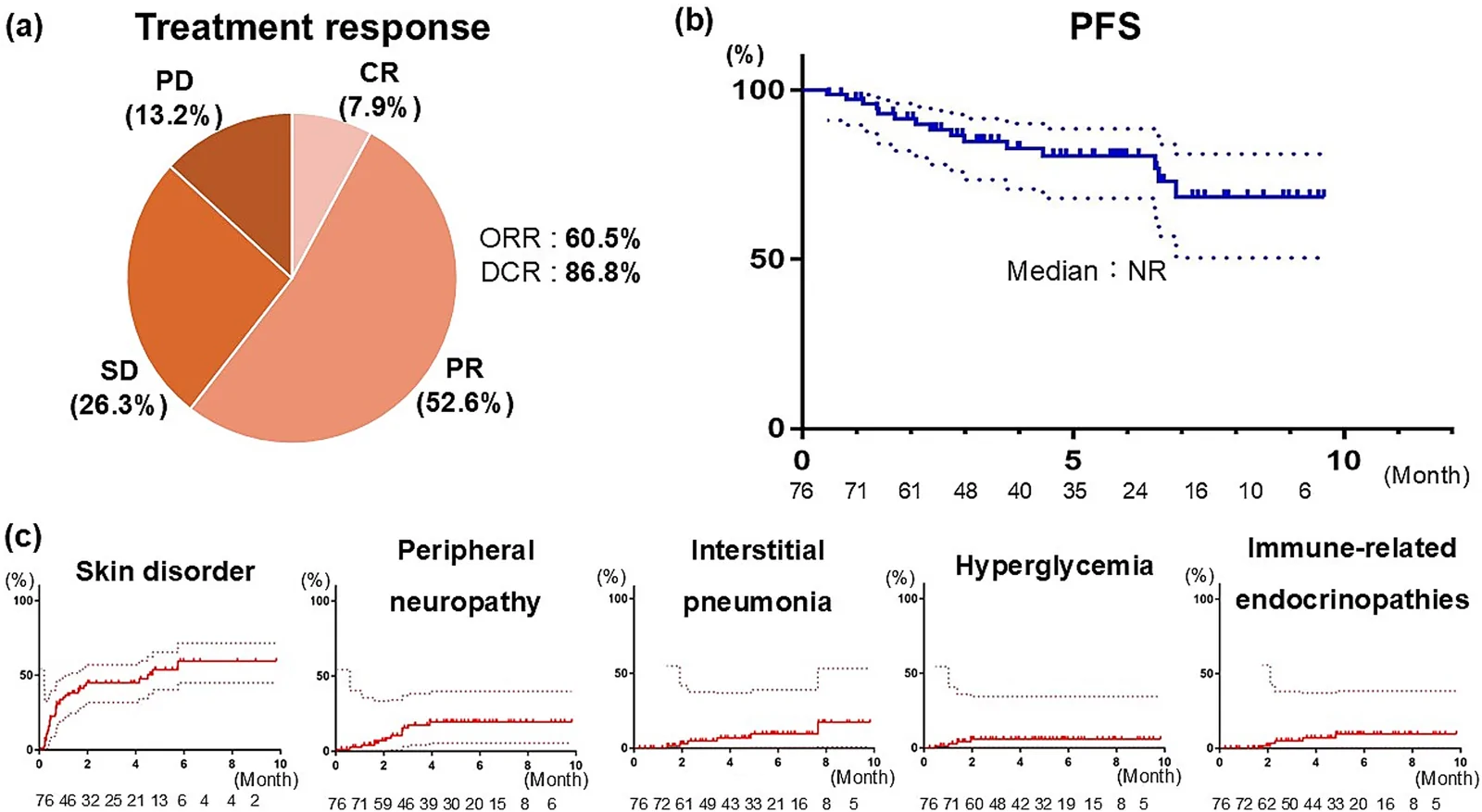
(a) The proportion of treatment responses to EV + P. ORR comprised CR and PR. DCR comprised CR, PR, and SD. (b) Kaplan–Meier curve of PFS. Six-month PFS was 80.6%. Median PFS was not reached. Numbers at risk are presented under the curve. (c) Kaplan–Meier curves of the cumulative incidence of each common AE after induction of EV + P treatment. Numbers at risk are presented under the curve. CR, complete response; DCR, disease control rate; EV + P, enfortumab vedotin plus pembrolizumab; NR, not reached, ORR, objective response rate; PD, progressive disease; PFS, progression-free survival; PR, partial response; SD, stable disease; AE, adverse event; irAE, immune-related AE.

PFS (Fig. 1b) and OS (Online Resource, Supplementary Fig. S1) were calculated after EV + P treatment initiation. Six-month PFS and OS rates were 80.6% and 85.1%, respectively. Median PFS and OS were not reached.

### AEs due to EV + P treatment

Table 2 presents a list of the observed AEs. The most frequently recorded AE was skin disorders, which occurred in 36 (47.4%) patients. Peripheral neuropathy, interstitial pneumonia, hyperglycemia, hypothyroidism, and adrenal insufficiency were observed in 11 (14.5%), 6 (7.9%), 4 (5.3%), 3 (3.9), and 2 (2.6%) patients, respectively. AEs of grade ≥ 3 included skin disorders in 8 (10.5%) patients, general malaise in 4 (5.3%), diarrhea in 2 (2.6%), interstitial pneumonia in 2 (2.6%), cytopenia in 4 (5.3%), hyperglycemia in 1 (1.3%), hepatic disorder in 1 (1.3%), and adrenal insufficiency in 1 (1.3%). No grade 5 AEs were recorded in this cohort.

**Table 2:** List of adverse events (*n* = 76).

	Any grade *n* (%)			Grade ≥ 3 *n* (%)			Grade ≤ 2 only *n* (%)		
	Total (*n* = 76)	Eligible (*n* = 35)	Ineligible (*n* = 41)	Total (*n* = 76)	Eligible (*n* = 35)	Ineligible (*n* = 41)	Total (*n* = 76)	Eligible (*n* = 35)	Ineligible (*n* = 41)
Skin disorders	36 (47.4)	18 (51.4)	18 (43.9)	8 (10.5)	4 (11.4)	4 (9.8)	28 (36.8)	14 (40.0)	14 (34.1)
Peripheral neuropathy	11 (14.5)	4 (11.4)	7 (17.1)	0 (0.0)	0 (0.0)	0 (0.0)	11 (14.5)	4 (11.4)	7 (17.1)
General malaise	10 (13.2)	3 (8.6)	7 (17.1)	4 (5.3)	1 (2.9)	3 (7.3)	6 (7.9)	2 (5.7)	4 (9.8)
Diarrhea	8 (10.5)	4 (11.4)	4 (9.8)	2 (2.6)	0 (0.0)	2 (4.9)	6 (7.9)	4 (11.4)	2 (4.9)
Taste disorders	8 (10.5)	5 (14.3)	3 (7.3)	0 (0.0)	0 (0.0)	0 (0.0)	8 (10.5)	5 (14.3)	3 (7.3)
Interstitial pneumonia	6 (7.9)	4 (11.4)	2 (4.9)	2 (2.6)	2 (5.7)	0 (0.0)	4 (5.3)	2 (5.7)	2 (4.9)
Cytopenia	6 (7.9)	2 (5.7)	4 (9.8)	4 (5.3)	2 (5.7)	2 (4.9)	2 (2.6)	0 (0.0)	2 (4.9)
Hyperglycemia	4 (5.3)	3 (8.6)	1 (2.4)	1 (1.3)	1 (2.9)	0 (0.0)	3 (3.9)	2 (5.7)	1 (2.4)
Hepatic disorders	3 (3.9)	0 (0.0)	3 (7.3)	1 (1.3)	0 (0.0)	1 (2.4)	2 (2.6)	0 (0.0)	2 (4.9)
Hypothyroidism	3 (3.9)	2 (5.7)	1 (2.4)	0 (0.0)	0 (0.0)	0 (0.0)	3 (3.9)	2 (5.7)	1 (2.4)
Adrenal insufficiency	2 (2.6)	2 (5.7)	0 (0.0)	1 (1.3)	1 (2.9)	0 (0.0)	1 (1.3)	1 (2.9)	0 (0.0)
Polyarthritis	0 (0.0)	0 (0.0)	0 (0.0)	0 (0.0)	0 (0.0)	0 (0.0)	0 (0.0)	0 (0.0)	0 (0.0)
Others	17 (22.4)	9 (25.7)	8 (19.5)	5 (6.6)	3 (8.6)	2 (4.9)	12 (15.8)	6 (17.1)	6 (14.6)

For CDDP eligibility, eligible was defined as eGFR ≥50.0 mL/min/1.73 m^2^ and PS ≤1.

CDDP, cisplatin; eGFR, estimated glomerular filtration rate; PS, performance status.

The cumulative incidence of each AE was calculated (Fig. 1c). In patients who experienced each AE, the median time to onset of skin disorders was 20 (IQR, 10–37) days, peripheral neuropathy was 66 (IQR, 37–86) days, interstitial pneumonia was 86 (IQR, 55–172) days, hyperglycemia was 37 (IQR, 19–55) days, and immune-related endocrinopathies was 71 (IQR, 60–128) days.

### Comparisons of the CDDP-eligible and CDDP-ineligible groups

Patient characteristics and treatment data were compared between the CDDP-eligible and CDDP-ineligible groups (Table 1). Patients were significantly older (*P* = .033), and the male-to-female ratio was significantly higher (*P* = .0496) in the CDDP-ineligible group compared with the CDDP-eligible group. The proportion of patients with primary tumors of the upper urinary tract (renal pelvis or ureter) in the CDDP-ineligible group was significantly higher (p = 0.007) than that in the CDDP-eligible group, which may be due to the higher likelihood of developing renal impairment induced by disease progression or treatment. No significant differences in the histological type, metastatic lesions, history of each treatment, or incidence of AEs were observed between the groups. A detailed list of the AEs in each group is provided in Table 2.

No significant differences in treatment response, ORR (68.6% vs. 53.7%), DCR (88.6% vs. 85.4%) (Fig. 2a), or PFS (Fig. 2b) were observed between the groups. Moreover, the cumulative incidence of common AEs (skin disorders, peripheral neuropathy, interstitial pneumonia, hyperglycemia, and immune-related endocrinopathies) did not differ between the groups (Fig. 3).

**Figure 2:**
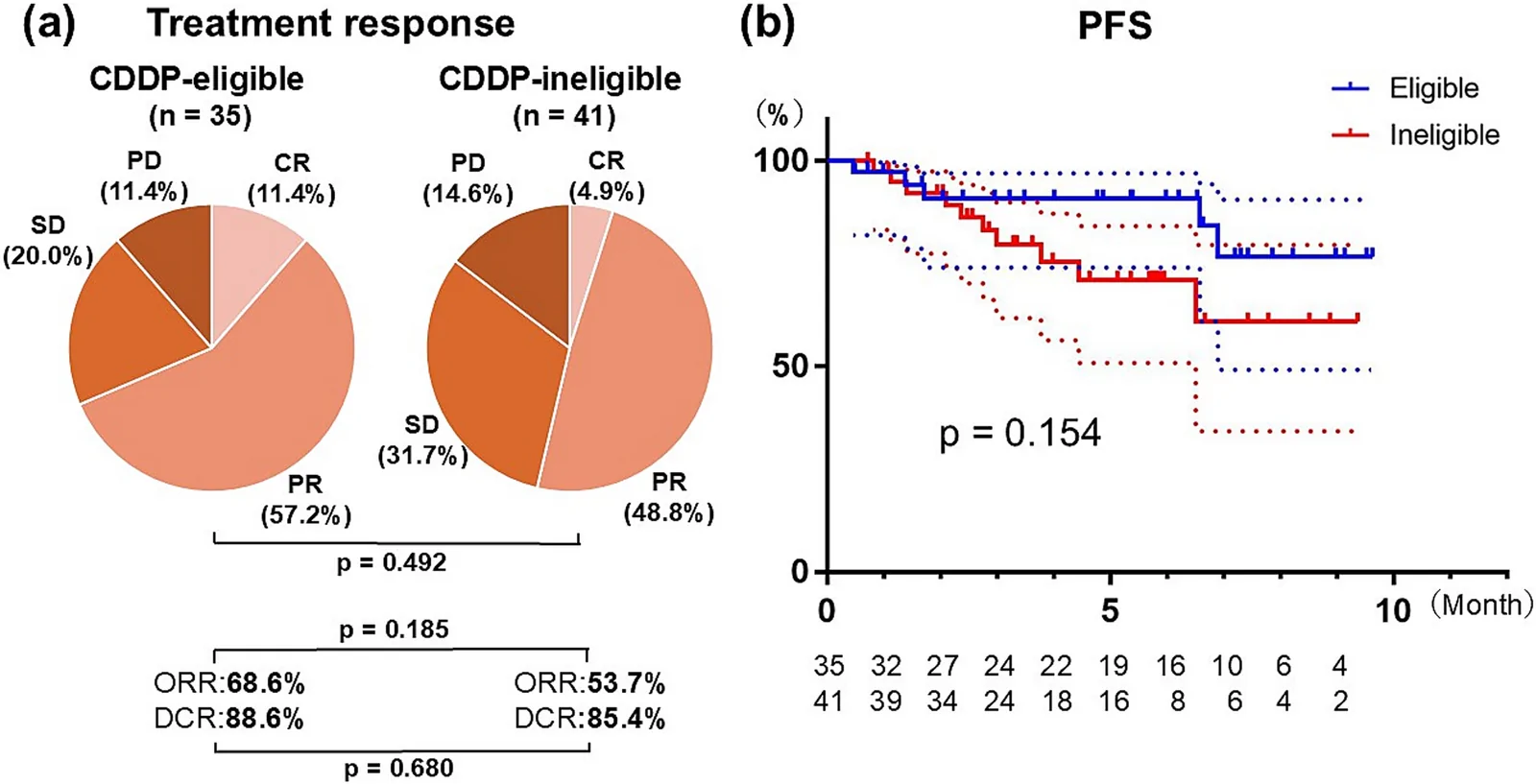
(a) The proportion of treatment responses, ORR, and DCR to EV + P treatment in CDDP-eligible and CDDP-ineligible patients. (b) A comparison of PFS between the two groups based on CDDP eligibility. Numbers at risk are presented under the curve. CDDP, cisplatin; CR, complete response; DCR, disease control rate; EV+P, enfortumab vedotin plus pembrolizumab; ORR, objective response rate; PD, progressive disease; PFS, progression-free survival; PR, partial response; SD, stable disease.

**Figure 3:**
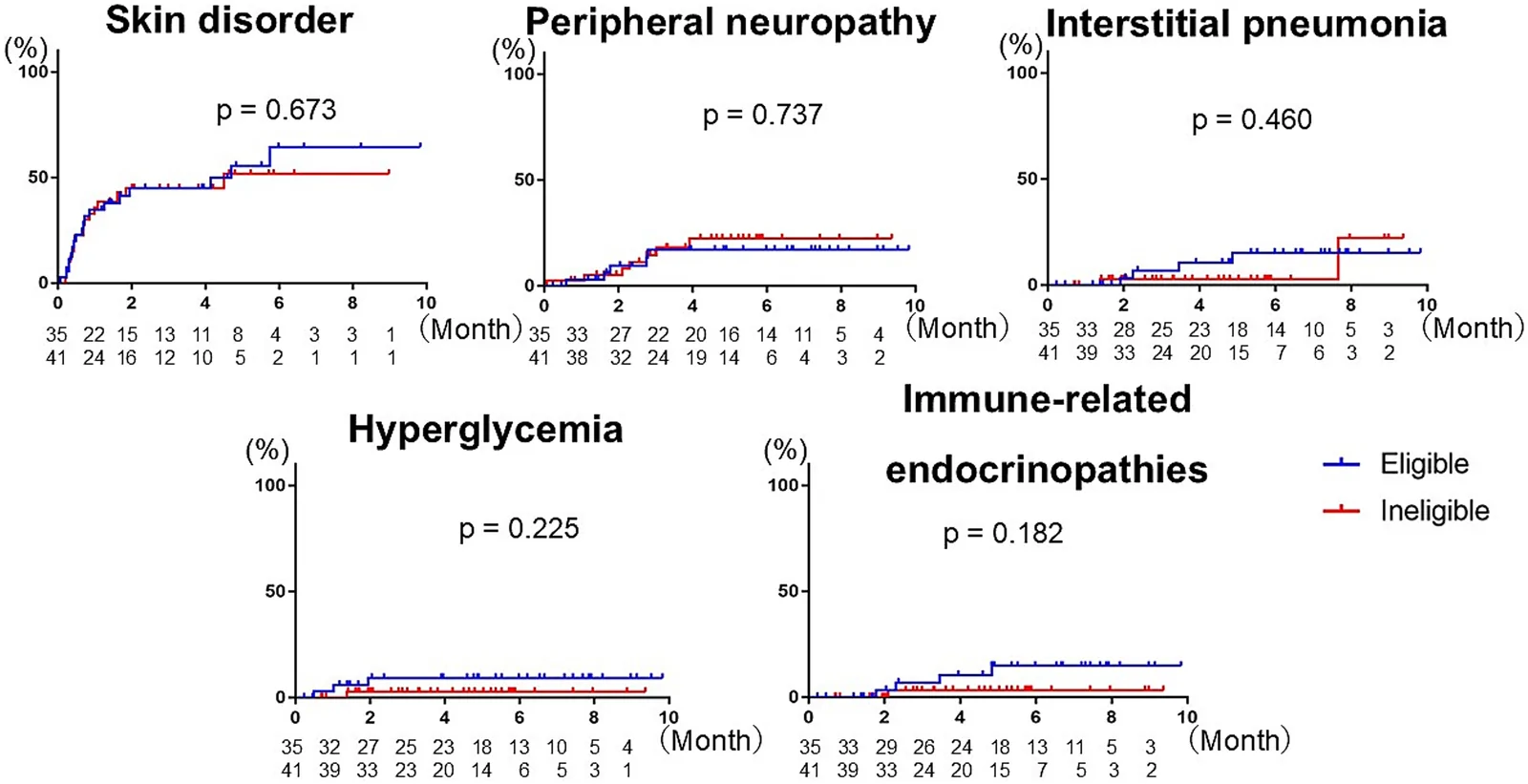
A comparison of the cumulative incidence of each common AE after induction of EV + P treatment between the two groups based on CDDP eligibility. Numbers at risk are presented under the curve. AE, adverse event; CDDP, cisplatin; EV + P, enfortumab vedotin plus pembrolizumab; irAE, immune-related AE.

Considering EV dose modifications, the median cumulative incidence was 4.87 months (Fig. 4a). The minimum EV dose was 1.25 mg/kg in 51.3% of patients, 1.0 mg/kg in 21.0%, 0.75 mg/kg in 5.3%, and 0 mg/kg in 22.4% (Supplementary Fig. S2a). The average minimum EV dose was 0.89 mg/kg excluding treatment discontinuation due to disease progression. No significant differences in the cumulative incidence of EV dose modifications were observed between the CDDP-eligible and CDDP-ineligible groups (Fig. 4b). The minimum EV doses for each group are shown in Supplementary Fig. S2b. No significant differences were observed between the groups in terms of the average minimum EV dose (0.86 vs. 0.92 mg/kg; *P* = .781).

**Figure 4:**
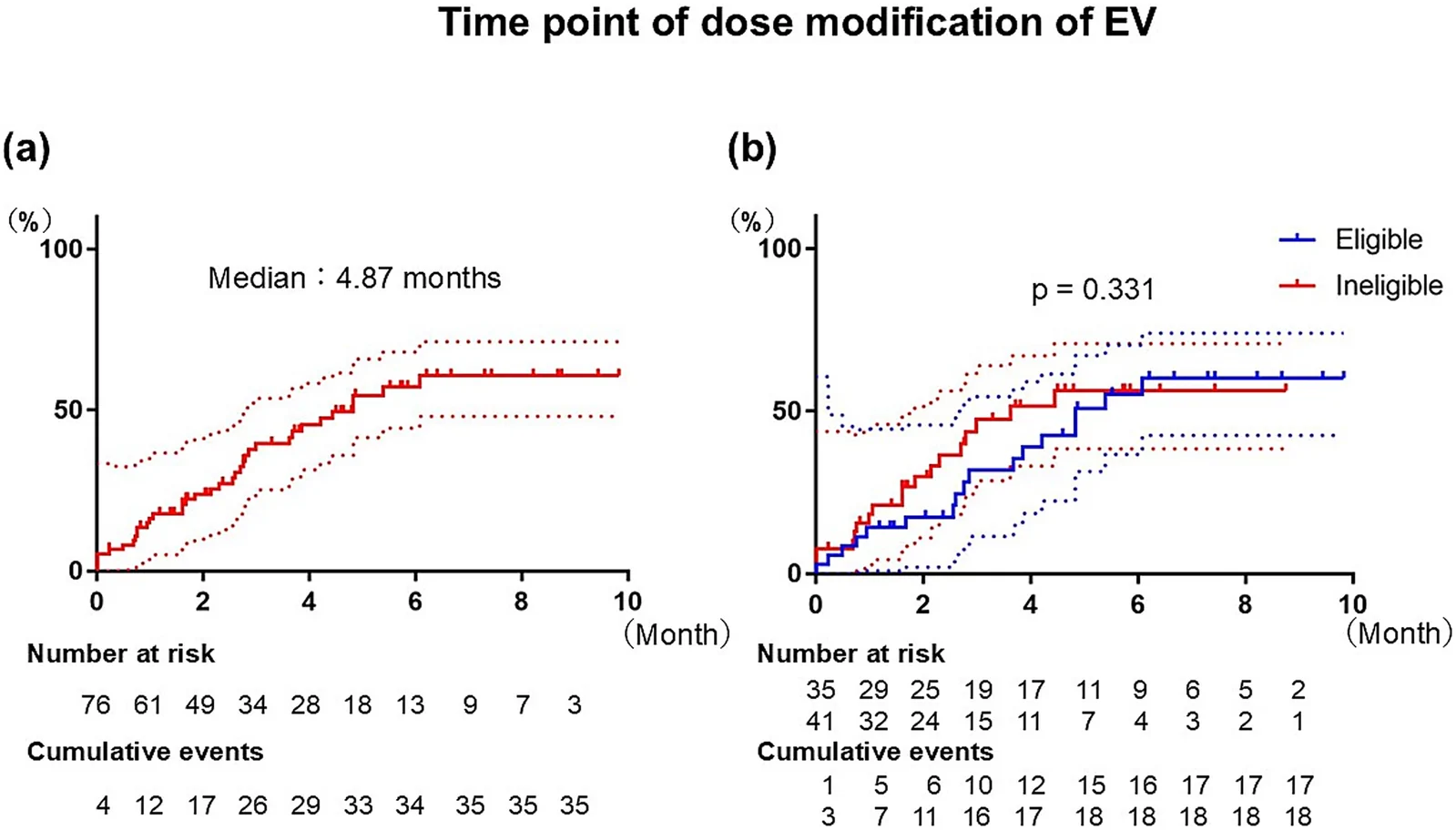
(a) Kaplan–Meier curve of EV dose modifications after induction of EV + P treatment. Median time point was 4.87 months. Numbers at risk and cumulative events are presented under the curve. (b) A comparison of the cumulative incidence of EV dose modifications between the two groups based on CDDP eligibility. Numbers at risk and cumulative events are presented under the curve. CDDP, cisplatin; EV, enfortumab vedotin, EV + P, enfortumab vedotin plus pembrolizumab.

## Discussion

We assessed the efficacy of EV + P treatment in the present study; the ORR and DCR were 60.5% and 86.8%, respectively, and the 6-month PFS and OS were 80.6 and 85.1%, respectively. These results seem comparable to previous reports in both clinical trial and real-world settings. Specifically, the ORR and DCR were 67.8% and 76.5%, respectively, in a clinical trial setting [7] and 73.3% and 82.6%, respectively, in a real-world report [8]. Six-month PFS and OS rates were approximately 75% and 90%, respectively, in the clinical trial setting and approximately 70% and >80%, respectively, in the real-world report.

During the median 5.56 months of EV + P treatment, AEs of any grade and grade ≥ 3 occurred in 77.6% and 32.9% of the patients in this study, respectively. These incidence rates were relatively lower than those reported previously [7–9]. In the clinical trial setting [7], AEs of any grade and grade ≥ 3 occurred in 97.0% and 55.9% of patients, respectively, during a median 9.4 months. In the real-world report [8, 9], AEs of any grade and grade ≥ 3 occurred in 91.7%/100.0% and 28.3%/39.6% of patients, respectively, during a median follow-up 14.0/2.5 months. The differences in incidence rates between this study and previous reports were likely due to differences in ethnic backgrounds or the observation period of each cohort. In particular, the incidence rate of peripheral neuropathy was lower in the current study than in previous reports (any grade/grade ≥ 3, 14.5%/0.0% vs. 50.0%/3.6% [7] and 39.9%/6.0% [8]). The reason of the result might be because of short follow-up. The rates of peripheral neuropathy in our study were also lower than those reported in a previous real-world cohort of late-line EV monotherapy with a median follow-up of 8 months (any grade/grade ≥ 3, 25.6%/9.6%) [10]. However, our observed rates were comparable with those of a Japanese cohort receiving EV monotherapy (any grade/grade ≥ 3, 14.3%/0.0%) [11], suggesting a lower incidence of peripheral neuropathy during EV administration in Japanese patients than in those with other ethnicities. However, no definitive conclusions can be made, as peripheral neuropathy frequently presents with a delayed onset [12]. Furthermore, grade 5 AEs did not occur in the current study, whereas some grade 5 AEs have been previously reported, including multiple organ dysfunction syndrome, diarrhea, asthenia, immune-related or interstitial lung disease and myocarditis, and sepsis [7–9]. Importantly, the time to onset of peripheral neuropathy, interstitial pneumonia, and immune-related endocrionopathies was ≥60 days in the current study, indicating that careful long-term follow-up may be required, despite an acceptable safety profile.

Efficacy and safety did not significantly differ between CDDP-ineligible and CDDP-eligible patients, which is consistent with a previous report in a clinical trial setting [13]. Specifically, the ORR was reported as 63.9% in the CDDP-ineligible group and 70.8% in the CDDP-eligible group, compared with 53.7% vs. 68.6% in the current study; furthermore, 32.5% and 24.7% of patients achieved CR in the CDDP-ineligible and CDDP-eligible groups, respectively, compared with 11.4% and 4.9% in the current study. Although GC plus nivolumab is also a first-line systemic treatment for patients with advanced UC, with better outcomes than conventional GC chemotherapy [14], this regimen cannot be administered to CDDP-ineligible patients. In contrast, EV + P treatment is indicated for almost all patients, regardless of CDDP eligibility.

Reducing the EV dose by 0.25 mg/kg is recommended after the occurrence of AEs that preclude continued administration at the same dose. This study demonstrated that more than half of patients receiving EV + P treatment without disease progression for 4.8 months continued EV treatment without dose reduction. To the best of our knowledge, no comprehensive studies on dose modifications of EV in EV + P treatment have been previously reported. In a study on advanced UC in American cancer clinics, Mamtani *et al.* reported that the median EV dose was 1.1 mg/kg during a median treatment duration of 5 months in a late-line EV monotherapy setting [15]. In this setting, patients received 1.25 mg/kg of EV on days 1, 8, and 15 of a 28-day cycle. More patients received EV treatment without dose reduction in the current study than in this previous EV monotherapy report, which was likely due to the better general condition of treatment-naïve patients or to the lower frequency of EV administration compared with late-line settings.

Although steroid premedication is not routinely mandated during EV monotherapy, emerging evidence suggests that it may help mitigate cutaneous toxicities without compromising antitumor efficacy [16, 17] while the influence on antitumor efficacy remains controversial [18]. Recent report suggested that routine steroid premedication might contribute to cutaneous toxicities maintaining efficacy also in EV + P treatment [19]. In this cohort study, 81.6% of total patients underwent EV + P treatment received steroid premedication.

Safety and real-world use of this combination treatment could provide more valuable data because the treatment is likely to be incorporated into perioperative treatment in the near future [20]. Although the precise mechanisms remain incompletely understood, EV + P treatment is thought to modulate the tumor microenvironment and augment antitumor immunity [21]. There is also limited data regarding the mechanisms of resistance to EV and the associated clinical predictors [22]. A better understanding of the mechanisms underlying the combination therapy, as well as biomarkers associated with treatment resistance, may contribute to improving the future management of this therapeutic strategy.

Several limitations should be acknowledged. First, this study had a retrospective design and limited patient information. Incomplete records or selection bias may have affected the data collection. DCR, which includes SD, might be affected by bias arising from the timing of radiological assessments. The observation period was too short, and sample size was too small to evaluate robust outcomes. Several AEs occurred too infrequently to permit robust statistical analysis. Minimum dose should not be considered a precise surrogate for relative dose intensity. Because follow-up duration differed significantly between the CDDP-eligible and CDDP-ineligible groups, comparisons between the two groups should be interpreted with caution. An imbalance in the distribution of primary tumor sites between the two groups may have introduced bias into the comparisons. Although stratified analyses according to primary tumor site and multivariable adjustment for potential confounders would have been desirable, these approaches were limited by the relatively small sample size. Lastly, the indicators for dose modifications depended on the physicians at each institution. Consequently, further studies with larger cohorts are required to validate our results.

In conclusion, the short-term efficacy of first-line EV + P treatment for patients with advanced UC seemed comparable to that in previous reports, and the safety profile was acceptable, regardless of CDDP eligibility.

## Supplementary Material

Fig_S1_hyag117

Fig_S2_hyag117

## Data Availability

All data generated or analyzed during this study are included in this published article and its supplementary information files.
